# Modulatory Effects of Antibody Replacement Therapy to Innate and Adaptive Immune Cells

**DOI:** 10.3389/fimmu.2017.00697

**Published:** 2017-06-16

**Authors:** Isabella Quinti, Milica Mitrevski

**Affiliations:** ^1^Department of Molecular Medicine, Sapienza University of Rome, Rome, Italy; ^2^Department of Clinical Medicine, Sapienza University of Rome, Rome, Italy

**Keywords:** common variable immune disorders, X-linked agammaglobulinemia, IVIg replacement, innate immunity, adaptive immunity, *in vivo*

## Abstract

Intravenous immunoglobulin administered at replacement dosages modulates innate and adaptive immune cells in primary antibody deficiencies (PAD) in a different manner to what observed when high dosages are used or when their effect is analyzed by *in vitro* experimental conditions. The effects seem to be beneficial on innate cells in that dendritic cells maturate, pro-inflammatory monocytes decrease, and neutrophil function is preserved. The effects are less clear on adaptive immune cells. IVIg induced a transient increase of Treg and a long-term increase of CD4 cells. More complex and less understood is the interplay of IVIg with defective B cells of PAD patients. The paucity of data underlies the need of more studies on patients with PAD before drawing conclusions on the *in vivo* mechanisms of action of IVIg based on *in vitro* investigations.

## Introduction

Polyvalent intravenous immunoglobulin (IVIg) are used as replacement therapy in patients with primary antibody deficiencies (PAD), a group of defects characterized by a failure to mount protective antibody responses, including X-linked agammaglobulinemia (XLA) and common variable immune disorders (CVID), and as immunomodulatory treatment with high IVIg doses in patients with inflammatory-autoimmune diseases. IVIg contain a broad spectrum of antibody specificities against microorganism antigens able to opsonize and neutralize microbes and toxins. IVIg also contain functionally relevant natural autoantibodies toward a wide range of self-motifs such as Siglec 9, Fas, and BAFF, together with a wide range of specificities including idiotypes of immunoglobulins, T cell receptor, HLA molecules, and other cell surface molecules of immunological importance such as CD4, CD5, BAFF, Fas, cytokines, cytokine receptors, and chemokine receptors that may participate in regulation of the immune response (1).

There are many reports on the various immunological effects of high-dose IVIg treatment in patients with inflammatory diseases. Clearly, the significantly smaller doses of immunoglobulin prescribed to PAD patients for replacement therapy may convey different effects (2). The possible immunomodulatory effect of IVIg administered at replacement dosages on innate and adaptive immune cells in patients with PAD needs to be addressed by detailed studies since the peak plasma IgG level reached in patients on replacement administration is much lower that the peak reached in patients with autoimmune-inflammatory disorders. Moreover, *in vitro* studies might not be a suitable system to replicate the *in vivo* effects of IVIg. In fact, it is possible that the *in vitro* effects of IVIg do not recapitulate the *in vivo* effects since many cellular and mediator interactions are lacking when IVIg are added *in vitro* to experimental conditions. Moreover, *in vivo* studies might help to analyze the immunomodulatory short- and long-term effects of immunoglobulin on immune cells and the beneficial effects due to the reduction of the infection-associated immune activation that is likely to occur as a result of immunoglobulin replacement.

Several theories have been postulated about the mechanisms through which IVIg preparations exert their immune-regulatory properties at replacement dosages possibly involving different type of cells acting in concert (3). Moreover, the diversity of CVID immunological and clinical phenotype could affect the results of some of the experiments. In addition, the commercial IVIg preparation used to study the *in vivo* or *in vitro* effect should be considered, in that IVIg consist mainly of monomeric IgG, but if a residual amount of dimers is present in the preparation, the biological effects might be different (4).

## Polymorphonuclear Neutrophils (PMN)

In response to pathogens, PMN rapidly migrate to the site of inflammation, release proteolytic enzymes and antimicrobial peptides as well as reactive oxygen species. IVIg might modulate PMN activity by a saturating and an activating/inhibiting effect on PMN FcγRs (5). Almost 20 years ago, the first demonstration that IVIg administered at low dosages in patients affected by PAD did not alter neutrophils functions was published (6). Phagocytosis, intracellular bactericidal activity, and chemotaxis of PMN in PAD patients treated at very low dosages (IVIg 200 mg/kg/month) and at replacement dosages (IVIg 600 mg/kg/month) were comparable to those of healthy controls (6). We have recently confirmed these data showing that in CVID and XLA patients, PMN were capable *in vivo* to perform efficient migration, degranulation, phagocytosis, and oxidative burst at baseline and shortly after IVIg administration (7, 8). Moreover, IVIg infusion-administered *in vivo* at replacement dosages did not alter the PMN expression of receptors involved in PMN functions, such as CD181, CD66b, CD11b, CD11c, CD16, and Siglec 9 (7, 8).

**Figure 1:**
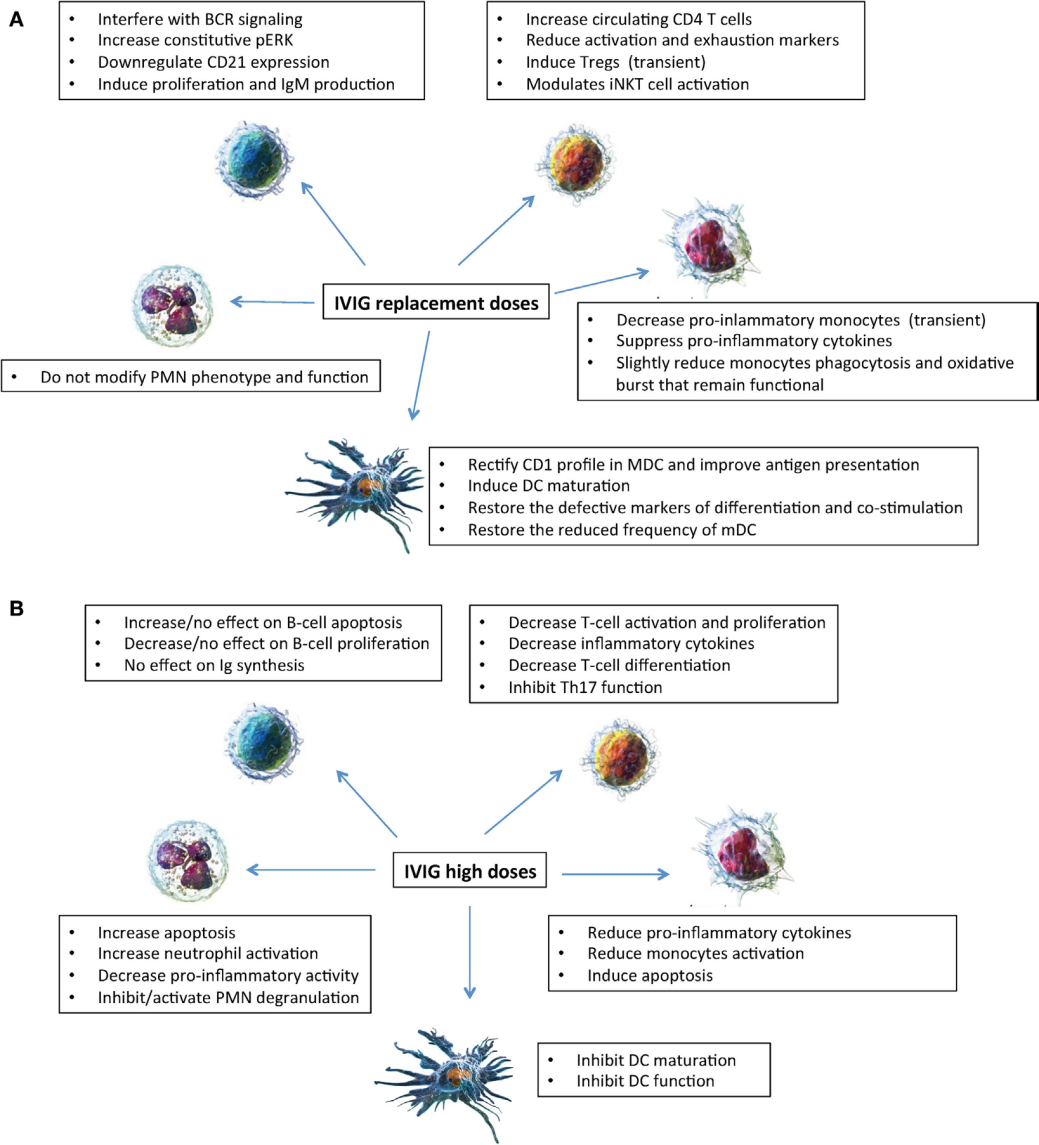
Mechanisms of action of intravenous immunoglobulin (IVIg) on innate (neutrophils, monocytes, and dendritic cells) and adaptive (B and T lymphocytes) immune cells. Effects of IVIg (replacement dosages) **(A)**; effects of IVIg (high dosages) **(B)**. Cell images from Ref. (55).

In contrast with the *in vivo* data obtained from CVID and XLA patients infused with IVIg, experiments performed with IVIg added *in vitro* on isolated PMN or to whole blood (9) showed that IVIg (1–25 mg/ml) might affect the overall activity of PMN by (1) inducing apoptosis; (2) decreasing the pro-inflammatory activity; (3) inhibiting or activating PMN degranulation (10–15) (Figure 1).

Thus, *in vitro* experiments provided conflicting results of immunomodulatory effects on PMN activity depending on the concentration of IVIg added *in vitro*, while *in vivo* data showed that PMN remained fully functional in patients treated with replacement IVIg dosages (Figure 1).

## Monocytes

Monocytes are now classified according to their expression of CD14, the receptor of LPS, and CD16, a low affinity Fcγ receptor, into three different subsets: classical monocytes, non-classical monocytes, and intermediate monocytes also called pro-inflammatory monocytes based on gene expression profiling and cytokine production, such as TNF-α, IL-1β, and IL-6 (16, 17). We have recently shown that CVID and XLA patients displayed an increased frequency of CD14^bright^CD16^+^ pro-inflammatory intermediate monocytes (8, 18). In CVID and XLA patients, shortly after IVIg infusion, we observed a transient reduction of about one-fourth of peripheral monocytes, a decrease involving mainly pro-inflammatory intermediate monocytes. These data are in agreement with other observations showing that IVIg infusion induced a transient decrease in the number of pro-inflammatory monocytes that returned at baseline levels after 20 h and suppressed the production of pro-inflammatory cytokines (19). These phenomena might be an additional mechanism through which IVIg infusion exert an anti-inflammatory effect, even when infused at replacement dosages. It is possible that an apoptotic process is involved. In fact, IVIg preparations contain agonistic and antagonistic anti-CD95 antibodies interacting with CD16^+^ monocytes that constitutively upregulated proapoptotic genes (20). Thus, these naturally occurring autoantibodies might contribute to the anti-inflammatory effects of IVIg *via* cell death regulation. In CVID and XLA shortly after IVIg infusion, we also observed a reduction of expression of functional monocyte receptors such as CD11b and Siglec 9 on classical monocytes associated, as expected, with a slight reduction of phagocytosis and oxidative burst functions that even if decreased remained in the normal range (8, 18). As described for anti-CD95, the reduction of Siglec 9 receptor might also be related to the binding of anti-Siglec 9 antibodies contained in IVIg preparations (20). It is then possible that IVIg bind to the Fc receptors on monocytes’ surface and transiently limit the availability to bind opsonized bacteria.

In conclusion as shown in Figure 1, taken together our results showed that in XLA and CVID patients IVIg exert *in vivo* a short-term anti-inflammatory effect and do not severely affect the monocytes ability to appropriately respond to pathogens (8).

## Dendritic Cells

Dendritic cells are professional antigen-presenting cells essential for priming immune response. When immature, DC capture antigen efficiently, but only mature DC are potent T-cell activators. Maturation process modifies the expression of MHC molecules, co-stimulatory molecules and cytokines. CD1 proteins are MHC I-like markers of differentiation of DC. The groups 1 (CD1a, CD1b, and CD1c) and 2 (CD1d) are involved in lipid antigen presentation to the T cells (21). There are diverse subsets of DC: monocyte-related DC, blood myeloid (mDC), and plasmacytoid DC (pDC), precursors of tissue and lymphoid DC (22). In the presence of GM-CSF and IL-4, it is possible to induce the production *in vitro* of monocyte-derived DC (MdDC) (23, 24). Different subsets of DC express distinct CD1 profiles, suggesting that microenvironment could regulate diverse antigen presentation pathways.

Common variable immune disorder patients have a reduction of both pDC and mDC subsets (25). Their MdDC display disturbed differentiation, maturation, and function (24). *In vitro* and *in vivo* experiments showed similar effects of IVIg on DC: the addition *in vitro* of IVIg at a concentration of 10 mg/ml to DC cultures of CVID patients partially restored the defective markers of differentiation (CD1a) and co-stimulation (CD80, CD86, and CD40) (26); a recent *in vivo* study in CVID demonstrated that after 6–12 months from initiation of replacement, IVIg therapy partially restored the reduced frequency of mDC, the altered expression of their co-stimulatory molecules and the CD4 T cell count (27).

Moreover, *in vitro* studies showed that different concentrations of IVIg added to MdDC influenced the expression pattern of CD1 molecules (28); similarly, *in vivo* studies found that IVIg therapy normalized the elevated levels of CD1a and CD1b on mDCs of CVID patients (29). By regulating CD1 expression pattern on DC, IVIg treatment affects the balance between CD1d-restricted antigen presentation to iNKT cells and group 1 CD1-restricted antigen presentation to T cells (28). Thus, by fine tuning of the CD1 antigen presentation pathways, IVIg could provide suitable activation signals to the immune system.

Also XLA patients have reduced DC myeloid subset (25). Moreover, it was suggested that their DC failed to differentiate and to mature because of a hypogammaglobulinemic environment (30). These abnormalities were partially restored by adding IVIg *in vitro* during the differentiation of DC from monocytes. Natural CD40 reactive antibodies are likely to exert this effect through an agonistic action on CD40 (30).

Taken together, as shown in Figure 1, IVIg at replacement dosages has a number of beneficial effects on DC differentiation and function as demonstrated by *in vivo* and *in vitro* studies, which are different from the effects of IVIg (0.15 mM) administered at high dosages that inhibited maturation and function of DC (24).

## B Cells

The B cell compartment is variably disturbed in patients with CVID. The total number of peripheral B cells is reduced in about 40–50% patients (31, 32). Class-switched memory B cells are reduced in 80–90% of CVID patients (31) often in association with defects of B cell receptor (BCR) activation (33) as well as the TLRs (34).

Therapeutic immunoglobulins interplay with B cells both directly, with surface receptors or intracellular molecules, and indirectly, influencing cytokines, survival factors or through other immune cells (35). Nevertheless, there is a shortage of studies that could unveil the effects of IVIg on B lymphocytes, particularly when administered at replacement dosages.

*In vitro* studies provided conflicting results. Graded concentration of IVIg (from low dose to high dose) demonstrated that IVIg did not affect the proliferative capacity of B cells, did not cause significant apoptosis of B cells, neither affected mRNA synthesis of both IgM and IgG (36). Other *in vitro* studies showed that IVIg at high dose (0.15 mM) can directly inhibit B cell activation and proliferation (37) and can induce apoptosis in B lymphocytes by inducing a functional silencing program similar to anergy (38, 39) (Figure 1).

A recent study demonstrated that upon stimulation with physiological IVIg concentration added *in vitro* to experimental conditions, B cells from CVID patients were capable to proliferate and produce IgM (40). The authors suggested that antibodies within IVIg preparations rectified the defective signaling of B cells provided by T cells and delivered T-independent signals. We confirmed a signal dysregulation intrinsic to B cells in a subgroup of CVID patients. CVID patients with expanded CD21low B cells have high constitutive ERK activation [BCR signaling pathway important for B cell anergy (41)], low responsiveness to TLR9 and BCR stimuli, defective calcium signaling (42, 43), and propensity to apoptosis. IVIg infusion administered *in vivo* transiently increased constitutive pERK, reduced the pERK increment induced by BCR cross-linking (44), and drove B cells to downregulate CD21 expression (45).

Thus, data on IVIg and B cells remained unclear and need to be elucidated. Moreover, the long-term consequences of IVIg on B cells of PAD patients have never been addressed due to the complex relationship between IgG and a number of factors that might contribute to the diverse B cell abnormalities observed over time in heterogeneous disorders like CVID.

## T Cells

A relative loss of T-cell function in many CVID patients has been widely demonstrated, including low circulating CD4 T cells, low naive CD4 T cells, low antigen-specific T cells, impaired proliferation, activation, and secretion of cytokines (46). Many CVID patients present a persistent low CD4 T cell count. For the group of CVID patients with persistently low CD4 counts, it has been suggested to change the PID classification from CVID to late-onset combined immune deficiency (47).

An elegant study addressed the modulation of T cells in CVID patients treated with IVIG on a long-term basis. Following IVIg initiation, CD4 counts increase in the majority of CVID patients and can reach normal levels in some cases (48). IVIg reduced the expression of activation and exhaustion markers on CD4 and CD8 T cells, which might remain elevated for up to 1 year from IVIg treatment initiation. This suggests that IgG replacement control with time infection-associated factors implicated in T cell chronic activation (27). The authors concluded that an early initiation of IgG replacement therapy in CVID patients may be beneficial to prevent T cell activation by switching off the inflammatory status. The same effect was demonstrated in *in vitro* studies when IVIg were added to cultures even at physiological concentrations (49).

In CVID patients, Tregs frequency (50) and function have been shown to be reduced, and this defect might have a role in the pathogenesis of CVID-associated autoimmune or inflammatory complications. IVIg administration modulates Tregs as demonstrated by the transient increase in Tregs 30 min after IVIg infusion (51). However, no sustained effect on Treg frequency was observed, and thus additional studies are needed to address the *in vivo* long-term effect on Treg and the clinical impact of this finding.

In addition, CVID patients show a reduction of iNKT cells (52), a subset that control bacterial and viral infections by recognizing endogenous and bacterial-derived glycolipids presented by CD1d molecules. In CVID, iNKT cells showed an elevated expression of markers of activation and exhaustion such as HLA-DR, CD161, and PD-1. IVIg treatment did not improve the frequency of iNKT cells but reduced CD161and PD-1 expression and possibly reduced iNKT cell activation and exhaustion (27).

As shown in Figure 1, these effects differ from the data obtained *in vitro* when higher IVIg concentrations (0.15 mmol/L) were added to experimental conditions showing suppression of T cell proliferation (53), of amplification of human Th17 cells (54) and of multiple key effector/inflammatory cytokines (49). Taken together, IVIg at replacement dosages have a beneficial short-lived effect on Tregs and a beneficial long-term effect on T cells and possibly on iNKT cells.

## Conclusion

IVIg administered at replacement dosages modulate innate and adaptive immune cells in PAD (Figure 1) differently to what observed when the effects of high dosages are evaluated. The effects seem to be beneficial on innate cells in that dendritic cells maturate, pro-inflammatory monocytes decrease, and neutrophil function is preserved. The effects are less clear on adaptive immune cells where a transient increase of Treg and a long-term increase of CD4 were demonstrated. More complex and less understood is the interplay of IVIg with defective B cells of PAD patients. The paucity of data underlies the need of more studies before drawing conclusions on the *in vivo* mechanisms of action of IVIg based on *in vitro* investigations. Moreover, should be noted that all studies presented refer to IVIg only and no data are available on *in vivo* studies of subcutaneous administration. Moreover, studies on SCIg might help to discriminate between short-term effect of IVIg administration due to the IgG peak reached at the time of the infusion and long-term effect due a constant IgG replacement.

## Author Contributions

IQ and MM contributed to this paper by designing the perspective, by contributing with experimental data, by analyzing the data in the literature, and by writing the manuscript.

## Conflict of Interest Statement

IQ received travel grant, grant for Advisory Board participation and research grant from Kedrion, Shire, Octapharma, Grifols, and CSL Behring. MM declares no conflict of interest.
